# An overview of heavy-atom derivatization of protein crystals

**DOI:** 10.1107/S2059798316000401

**Published:** 2016-03-01

**Authors:** Ashley C. W. Pike, Elspeth F. Garman, Tobias Krojer, Frank von Delft, Elisabeth P. Carpenter

**Affiliations:** a Structural Genomics Consortium, University of Oxford, Roosevelt Drive, Oxford OX11 9HP, England; b Department of Biochemistry, University of Oxford, South Parks Road, Oxford OX1 3QU, England; c Diamond Light Source Ltd, Harwell Science and Innovation Campus, Didcot OX11 0QX, England; d Department of Biochemistry, University of Johannesburg, Aukland Park 2006, South Africa

**Keywords:** derivatization of crystals, heavy atoms, protein crystals, phasing techniques, crystallography

## Abstract

Heavy-atom derivatization is one of the oldest techniques for obtaining phase information for protein crystals and, although it is no longer the first choice, it remains a useful technique for obtaining phases for unknown structures and for low-resolution data sets. It is also valuable for confirming the chain trace in low-resolution electron-density maps. This overview provides a summary of the technique and is aimed at first-time users of the method. It includes guidelines on when to use it, which heavy atoms are most likely to work, how to prepare heavy-atom solutions, how to derivatize crystals and how to determine whether a crystal is in fact a derivative.

## Abstract 2

This review summarizes the reasons why the heavy-atom derivatization of protein crystals can be useful, how to select heavy atoms, how to produce a heavy-atom-modified crystal that still diffracts and how to determine whether the protein has been modified.

## 1. Introduction   

### 1.1. Why do you need to derivatize crystals? The phase problem   

Currently, protein crystallography is the most successful technique for determining the three-dimensional structures of proteins and other macromolecules. Ideally, it would be possible to collect a single data set from a native crystal and immediately calculate a map without any additional information. Unfortunately, however, there is a complication in structure determination, namely the ‘phase problem’, *i.e.* the fact that in a diffraction experiment the intensities of reflections are recorded but the phase information is lost.

To calculate the electron density ρ at a point (*x*, *y*, *z*) in a unit cell with volume *V*, for each *h*, *k*, *l* reflection both the structure-factor amplitudes |*F*_*hkl*_| (which are the square root of the intensities *I*_*hkl*_) and the phases α_*hkl*_ are required,
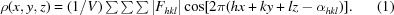



The above equation applies in cases of non-anomalous scattering where Friedel’s law is obeyed. Therefore, once the first X-ray diffraction data set has been collected, an electron-density map cannot be calculated without also obtaining an approximation for the phases.

### 1.2. Methods for obtaining phases without additional experimental information: molecular replacement and *ab initio* phasing   

**Figure 1:**
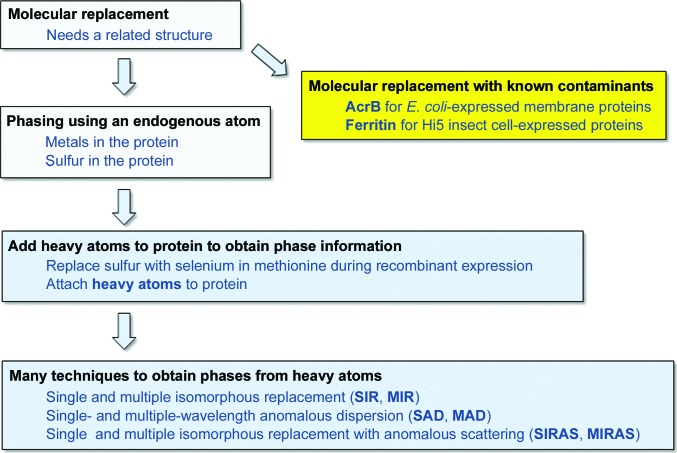
Techniques for the phasing of macromolecular structures.

A range of techniques are available for solving the phase problem (Fig. 1 ▸). The most commonly used method is molecular replacement (MR), an algorithmic approach that uses the structure of a related protein, positioned within the unit cell of the new crystal, to obtain approximate initial phases. There are now relatively few protein domain folds for which there are no known homologues, and the ever-increasing sensitivity of the algorithms (DiMaio *et al.*, 2011 ▸; Terwilliger *et al.*, 2012 ▸; Bunkóczi *et al.*, 2015 ▸), as well as new approaches to extend the range of the MR technique using automated *ab initio* modelling, have greatly increased the number of structures that can be solved by MR.

In cases where molecular replacement has failed, the possibility that the protein in the crystal is not the intended target should be considered. Crystallization of a low-abundance contaminating protein is a common occurrence, so it is worth checking for this problem before spending time on heavy-atom (HA) phasing. A quick way to identify known contaminants is to compare the unit-cell constants of the new crystal against the unit cells of all structures in the PDB (Ramraj *et al.*, 2012 ▸) and then attempt molecular replacement with proteins from crystals with similar unit cells. There is a particularly pernicious contamination problem with membrane proteins expressed in *Escherichia coli* and purified using a His tag. The *E. coli* protein AcrB often co-purifies and crystallizes readily, even when it is only 1% of the purified protein (Veesler *et al.*, 2008 ▸). Similarly, when expressing proteins in Hi5 insect cells from *Trichoplusia ni*, there is a secreted ferritin that can co-purify and give crystals easily (Hamburger *et al.*, 2005 ▸). It is of course unfortunate to discover that a contaminating protein has crystallized instead of the target protein, but it is better to know the enemy, and this problem is all too common, particularly for membrane proteins.

If the diffraction data are obtained to better than 1.2 Å resolution, then it may be possible to solve the structure from a single data set by using the *ab initio* direct-methods approaches developed for small-molecule crystallography (Usón & Sheldrick, 1999 ▸).

Attempts at *ab initio* phasing with small generic fragments and 2.0 Å resolution data sets have been successful in some cases using the program *ARCIMBOLDO* (Rodríguez *et al.*, 2012 ▸; Sammito *et al.*, 2014 ▸). Ensembles of *ab initio* models obtained from *ROSETTA*/*QUARK* have also been fruitful for lower resolution data sets using the program *AMPLE* (Bibby *et al.*, 2012 ▸). However, in many cases crystals do not give high enough resolution diffraction for these methods to work, particularly if they are being applied to challenging structures such as large complexes or membrane proteins.

Owing to its power and ease of use, MR enjoys great popularity, especially amongst novices in protein crystallo­graphy. However, while the basic concept is easy to understand, and setting up calculations is straightforward through graphical user interfaces in *CCP*4 (Winn *et al.*, 2011 ▸) and *PHENIX* (Adams *et al.*, 2010 ▸), the underlying mathematics is complex and it is not easy to troubleshoot when a solution is not forthcoming. In addition, with low-resolution data there is the issue of model bias, a phenomenon in which the phases from the model cause features to appear in an electron-density map that are derived from the search model but that are not actually present in the structure to be determined (Ramachandran & Srinivasan, 1961 ▸; Terwilliger *et al.*, 2008 ▸).

In contrast, although in general experimental phasing techniques (and especially HA-based phasing methods) are often used as a last resort, the actual experiments are simple and straightforward to evaluate. Experimental phasing methods provide unbiased phases that allow the final model to be obtained rapidly, accurately and with relatively few complications. In particular, for low-resolution structures, experimental phasing is essential to avoid model bias and errors in the assignment of the residues to the backbone. In these cases it can be more time-consuming to obtain and build marginal molecular-replacement solutions than to prepare, collect and analyse a HA soak. Different automated pipelines for experimental phasing are now available [*AutoSol* (Terwilliger *et al.*, 2009 ▸), *autoSHARP* (Vonrhein *et al.*, 2007 ▸) and *CRANK*2 (Skubák & Pannu, 2013 ▸)] which make the procedure almost as simple as molecular replacement, and since the phases from experimental phasing are not biased by a search model, model building and refinement will usually be straightforward.

### 1.3. Experimental phasing methods using HAs   

For proteins for which there are no known homologues for using MR and the crystals do not give high-resolution data, initial phases have to be obtained experimentally. This can be achieved if one or more HAs are present in an ordered arrangement in each asymmetric unit in the crystal. A HA is any atom with more electrons than those normally found in proteins (hydrogen, carbon, nitrogen, oxygen and sulfur). X-rays are diffracted by the electron cloud surrounding the nucleus in atoms, so the more electrons that there are in an atom, the more X-rays will be scattered. Thus, the higher the atomic number of the HA, the larger the number of electrons it will have and the greater the change in the diffraction pattern compared with the native crystals, so the easier it will be to obtain phases.

The change in diffraction amplitude (Δ*F*) with the addition of a HA was estimated by Crick & Magdoff (1956 ▸) as 〈Δ*F*〉/〈|*F*|〉 = *Z*_H_/*Z*_eff_ × (2*N*_H_/*N*_P_)^1/2^, where *Z*_H_ and *Z*_eff_ are the atomic numbers of the HA and the average atomic number for protein atoms (approximately 6.7), respectively. *N*_HA_ and *N*_P_ are the number of heavy atoms and non-heavy protein atoms, respectively. As an example, for a 100 kDa protein one fully occupied U atom (*Z* = 92) site would give an average change in amplitude of 20%, whereas a Cu atom (*Z* = 28) site would give only a 5.6% change.

Once ordered HAs are attached to the protein in defined locations within the crystal, then either one or several data sets with and without attached HAs can be compared (single or multiple isomorphous replacement; SIR or MIR, respectively) or one or more data sets can be collected at or near one of the X-ray absorption edges of the HA [a wavelength where Friedel’s law (*F*_*hkl*_ = *F*_−*h*, −*k*, −*l*_) breaks down] and the differences between the Friedel pairs (the so-called ‘anomalous signal’; AS) can be used to calculate the initial phases. This involves techniques called single-wavelength anomalous dispersion (SAD) or multiple-wavelength anomalous dispersion (MAD) depending on whether data are collected at a single (SAD) or multiple (MAD) wavelengths. A combination of both methods is also feasible (single or multiple isomorphous replacement with anomalous dispersion; SIRAS or MIRAS, respectively). Modern algorithms have made these distinctions somewhat academic because they can incorporate multiple types of signal simultaneously when calculating phase estimates.

Recently, it has also become possible to solve structures using the anomalous signal from the native S atoms in methionines and cysteines in a protein (Hendrickson & Teeter, 1981 ▸; Debreczeni *et al.*, 2003 ▸; Sarma & Karplus, 2006 ▸; Goulet *et al.*, 2010 ▸), although sulfur would not normally be regarded as a HA. The development of long-wavelength beamlines at synchrotrons (such as I23 at the Diamond Light Source; Wagner *et al.*, 2016 ▸) specifically designed for sulfur phasing will further increase the popularity of this method. In the end, however, it is likely that a number of lower resolution and larger structures will still require the HA derivatization of crystals to provide phases so that a map can be calculated and the structure can be solved.

### 1.4. Additional applications for HA phasing techniques   

An important use of HA derivatization of protein crystals is to aid the mapping of positions of residues in an electron-density map. Often with low-resolution data it is difficult to determine which part of the sequence belongs to which density. HAs that modify cysteine residues, or the use of selenomethionine in place of methionine, can provide useful additional information to define which residues belong where in the map. In extreme cases, where the resolution is in the range of 4 Å and the chain trace is unclear (*e.g.* the MATE transporter structure; He *et al.*, 2010 ▸), it may be necessary for a series of cysteine residues to be mutated into the protein which can then be modified by covalent binding to mercury, thus allowing the sequence to be assigned to the structure.

Another increasingly important use of HAs in crystals comes from the need to identify binding sites for small molecules in low-resolution structures. In these cases the small molecule can be modified to include a HA such as bromine or iodine, and can then be co-crystallized with, or soaked into, the crystal (Bagnéris *et al.*, 2015 ▸; Dong *et al.*, 2015 ▸). Although this can help with phasing the data set, it is more often used for locating the binding sites for the small molecule in the structure in cases where the resolution is low and/or the occupancy is limited.

Another use for data sets from HA-derivatized crystals is as an aid when triaging MR solutions. If phases have been obtained from a correct MR solution then there should be clear peaks in an anomalous difference Patterson map using the phases from the MR solution. Thus, the HA derivative can be used to assess the quality of MR solutions.

## 2. Which HAs to use  

Some proteins are born with heavy atoms (selenomethionine labelling), some achieve heavy atoms (cofactors or intrinsic metal ions) and some have heavy atoms thrust upon them (heavy-atom labelling)

(with apologies to W. Shakespeare).

The usefulness of a HA compound to derivatize a crystal will depend on a number of factors including whether HAs can be persuaded to bind to the protein and whether the crystal will diffract once the HA is attached. There are many excellent reviews of the subject of HA derivatization, including Blundell & Johnson (1976 ▸), Boggon & Shapiro (2000 ▸), Garman & Murray, (2003 ▸) and Lu & Sun (2014 ▸). Useful websites and databases are listed at the end of §[Sec sec2]2.

### 2.1. Endogenous metal ions   

The simplest way to obtain a protein with a HA attached is to select a protein that already has a HA bound when it is produced in a cell. More than 30% of all proteins fall into this category. These metal ions are important structural and/or functional components of the protein (reviewed in Harding *et al.*, 2010 ▸). The most common metals found in proteins are Na, Mg, K, Ca, Mn, Fe, Co, Ni, Cu and Zn. They are present either as cations directly bound to the protein or as part of a cofactor such as haem (which binds iron), chlorophyll (which binds magnesium), cobalamin (which binds cobalt) and the molybdopterin cofactors (which bind molybdenum or tungsten). Na and Mg are not particularly useful for phasing because they do not have a significant anomalous signal at any useful incident X-ray wavelength. However, in some crystals Mg can be replaced by Mn, thus providing an appreciable anomalous signal. K and Ca both have a significant anomalous signal, which is higher than that of S, and they all give an ever-increasing signal above 1.5 Å wavelength. Mn, Cu, Fe, Co and Ni all have *K* absorption edges between 1.28 and 1.9 Å. Thus, these endogenous HAs can be used for phasing without the need to add additional heavy metals. In addition, fluorescence scans around the anomalous edges for particular HAs can be used to characterize the metals present in a protein crystal, as a peak in the fluorescence signal is characteristic of specific HAs.

### 2.2. Substitution of selenomethionine for methionine   

Replacement of S atoms with selenium is now the most commonly used method for the modification of proteins with HAs (Hendrickson *et al.*, 1990 ▸; reviewed by Walden, 2010 ▸; Metanis & Hilvert, 2014 ▸). This works well with proteins that are expressed in *E. coli*, but is also feasible for proteins produced in other systems (Cronin *et al.*, 2007 ▸; Nettleship *et al.*, 2010 ▸). In *E. coli* expression systems, the methionine-auxotroph *E. coli* strain B834 (DE3) is grown on minimal medium with selenomethionine as the only source of methionine. Any protein expressed in such a system will have a large proportion of its methionines replaced by selenomethionines. The extent of substitution can be confirmed by mass spectrometry or proton-induced X-ray emission (Garman & Grime, 2005 ▸). Selenomethionine-labelled protein can also be produced in insect cells, although there are no methionine-auxotroph insect-cell strains available, so substitution relies on replacement of the medium with minimal medium containing selenomethionine, leading to incomplete substitution (40–70%; Bellizzi *et al.*, 1999 ▸). There is considerable variability in the expression levels, stability, crystallizability and diffraction quality of selenomethionine-labelled proteins compared with the wild-type protein, so although selenomethionine labelling is often the first derivatization method attempted, it is not guaranteed to work in all cases.

Selenium only has 34 electrons, compared with, for example, 80 electrons for mercury, so it provides lower X-ray scattering power per HA. Proteins with a large number of methionines and/or many copies of the protein in the asymmetric unit will have a correspondingly large number of Se atoms per asymmetric unit in the selenomethionine derivative. In these cases it can be difficult to determine the positions of the Se atoms. In addition, the anomalous scattering from selenium is relatively weak, with *f*′′ (a measure of the anomalous scattering from an atom at a given wavelength) being only 3.85 e^−^, compared with 10.19 e^−^ for Hg at their respective *K* and *L*_III_ absorption edges. For weaker reflections the small differences between the Friedel pairs may be masked by noise. Thus, for selenomethionine-labelled crystals that diffract to medium or low resolution the identification of Se sites may not be possible without additional information and the phasing power may be too low to provide useful phase information (Liu *et al.*, 2012 ▸). However, in these cases an initial HA derivative may allow the identification of selenium sites in selenomethionine-derivatized protein crystals, which can be useful for improving phasing and/or for tracing the chain.

One feature of using selenomethionine is that, depending on the protein and the size of the unit cell, a particular system may have very large numbers of heavy atoms that must be located in the course of phasing. In general this can be a major hurdle, although in favourable cases the power of direct methods [*SHELXD* (Schneider & Sheldrick, 2002 ▸), *SnB* (Xu *et al.*, 2008 ▸) and *HySS* (Grosse-Kunstleve & Adams, 2003 ▸)], combined with the fact that the Se atoms are typically well ordered, has led to very large systems being solved, *e.g.* 160 selenium sites for ketopantoate hydroxymethyltransferase from *E. coli*, which crystallized with two decamers in the asymmetric unit (von Delft *et al.*, 2003 ▸). On the other hand, once located, these sites deliver superior phasing power to the phasing calculation, yielding excellent maps; as a rule of thumb, the poorer the resolution and the larger the protein, the more selenium sites are required for successful phasing.

### 2.3. Classic methods of heavy-atom derivatization   

The classic method for derivatizing protein crystals involves a soaking experiment in which a metal-containing compound is added to a crystallization drop with preformed crystals. As an alternative, the protein can be mixed with a HA solution and incubated for some time and the excess HA can then be removed (using a buffer-exchange column or a concentrator), followed by crystallization as for the native protein.

The interaction between a protein and a HA depends on a complicated set of parameters, including the identity of the HA, its charge state, the ligands coordinating the metal, the available protein side chains and backbone atoms, the other components of the crystallization solution, and physical conditions such as the temperature.

A wide range of elements in many complexed forms have been tried in attempts to obtain phases for difficult structures. In order to form a derivative, the HA or a complex between a HA and a set of ligands has to preferentially bind to either side chains or backbone atoms in a protein, rather than bind to the ligands with which it was purified or other components of the solution (buffers, salts, water *etc.*). Ligands can be classified into two groups: ‘hard’ and ‘soft’ (Pearson, 1963 ▸; Blundell & Johnson, 1976 ▸). Hard ligands are electronegative and form electrostatic interactions without delocalization of electrons or the formation of covalent bonds. Examples of hard ligands are fluoride, water, glutamate, aspartate, carboxylate and the hydroxyls of serine and threonine. In contrast, soft ligands are polarizable, allowing delocalization of electrons and formation of covalent bonds. Examples of soft ligands are chloride, bromide, iodide, cyanide and imidazole. Side chains in proteins that act as soft ligands include cysteine, cystine, histidine and methionine.

**Figure 2:**
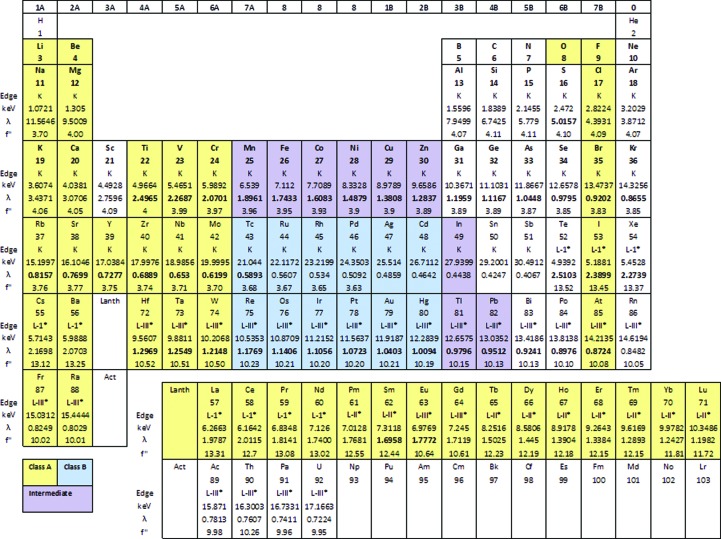
The periodic table with class A and class B heavy atoms shown in yellow and blue and intermediate elements in purple (adapted from Fig. 8.4 in Blundell & Johnson, 1976 ▸). The absorption edges (energies and wavelengths) and *f*′′ at the edge are shown (data from the http://skuld.bmsc.washington.edu/scatter/AS_periodic.html website). Some elements (marked with an asterisk) have a range of absorption edges from which to choose and thus for each element an edge at a suitable energy for data collection at a synchrotron has been selected.

HAs can be classified into Class A or Class B elements according to their ability to interact with hard or soft ligands, respectively (Fig. 2 ▸).

#### 2.3.1. Class A elements   

Metal ions on the left of the periodic table are generally class A and they form electrostatic interactions with hard ligands. They include the alkali metals, alkaline earths, lanthanides, actinides and transition metals. They form interactions with hydroxyls and carboxylates in proteins, with water and with buffer atoms such as acetate, citrate or phosphate.

#### 2.3.2. Class B elements   

In contrast, metal ions, particularly transition metals on the right of the periodic table, belong to class B (Fig. 2 ▸), forming covalent bonds with soft ligands. They include platinum, gold, silver and mercury, which can form covalent anionic complexes such as Pt(CN)_4_^2−^ and HgI_4_^−^. The metal ions form bonds with Cys, Met and His residues, with amino groups and with chloride and ammonia ions in buffer solutions. A particularly important example of this type of interaction is the formation of a covalent bond between mercury ions and cysteine residues. If a protein lacks cysteines, it may be helpful to insert a series of cysteine mutations in a range of locations for mercury derivatization to facilitate phasing and chain tracing. It is always worth preparing a range of mutations as several of them may fail to express. In the middle of the transition-metal series there is a range of ions that are intermediate between class A and class B (Fe^2+^ < Co^2+^ < Ni^2+^ < Cu^2+^ < Zn^2+^ in order of increasing softness). These ions can interact with either soft or hard ligands.

For each HA, there is a series of compounds available that can be tested for derivatization. The stability of the complex between a HA and its ligands is an important determinant of the behaviour of the HA. Table 1 ▸ shows examples of mercury compounds that are commercially available and have been successful in derivatization. There are two databases, HAD (Islam *et al.*, 1998 ▸) and HATODAS II (Sugahara *et al.*, 2009 ▸), that list a range of HA derivatives and their binding sites; links to these databases are given in §[Sec sec2.9]2.9 below.

#### 2.3.3. Factors affecting the usefulness of a HA   

A major factor affecting the utility of a HA is its interactions with the components of the crystallization mother liquor. Many heavy-metal salts are highly insoluble; for example, phosphate and sulfate buffers will lead to the formation of insoluble compounds with a wide range of metals. High concentrations of citrate or acetate can also precipitate or chelate certain metals, thus reducing their effective concentration. There is further information on the solubilities of individual HA salts in Chapter 8 of Blundell & Johnson (1976 ▸) and on the following website: http://xray0.princeton.edu/~phil/Facility/heavy_atoms.html.

High salt concentrations can also mask potential binding sites on proteins, blocking binding of the HA because the site is already occupied by the buffer cations in the crystallization solution.

Buffer components can also competitively displace the ligands surrounding metal ions, forming a more stable complex with the HA. If the affinity of the buffer for the metal is higher than for the available protein side chains, the HA will not bind to the protein.

In general, if the protein crystallizes in phosphate, ammonium sulfate or high concentrations of another salt, then it may be necessary to change the precipitant or move the crystal into a different precipitant before HA derivatization will be successful. This is less of a problem if the crystals are grown in polyethylene glycol (PEG) or an alcohol.

The pH of the crystallization solution can also have a significant effect on crystal derivatization both by changing the state of the protein side chains and by affecting the metal and its ligands. Some HA ions are insoluble at certain pH values, and lanthanides are not usable at higher pHs as they form gels. The pH can also affect the charge state of the protein side chains and termini. The p*K*_a_ for the C-terminal carboxylate is ∼3.0, that for Asp is 3.8, that for Glu is 4.3, that for His is 6.0, that for Cys is 8.3 and that for the N-terminal amine is ∼8, while Tyr, Lys and Arg all have p*K*_a_ values above 10. However, these values can be substantially modified depending on the local environment of the group. Thus, the use of the correct pH can be critical for successful derivatization.

If the intention is to collect anomalous difference data at a range of wavelengths around the absorption edge (SAD, MAD, SIRAS and MIRAS), access to a tuneable X-ray source will be advisable so that a convenient X-ray absorption edge for the HA can be reached. This is useful but not essential, since all heavy metals have an appreciable anomalous signal at incident X-ray energies below 15.5 keV (*i.e.* above a wavelength of 0.8 Å). Therefore, anomalous differences can be detected even if there is no absorption peak for the particular HA within the range of available X-ray energies.

#### 2.3.4. Systematic studies of heavy-atom derivatization   

In order to define a more systematic approach to HA derivatization, Boggon and Shapiro analysed the most successful HA compounds for derivatizing soluble proteins (Boggon & Shapiro, 2000 ▸). They identified the ‘Magic Seven’ shown in Table 2 ▸. Poul Nissen’s group analysed successful compounds for membrane-protein derivatization and identified the ‘Membrane’s Eleven’ list (Morth *et al.*, 2006 ▸; Table 3 ▸). This analysis was extended to include a further five compounds by Parker & Newstead (2013 ▸).

A systematic analysis of the reactivity of 40 HA compounds with peptides containing a single reactive side chain was completed by Peter Sun’s group. They tested the reactivity in a range of buffers and pH conditions using mass spectrometry to detect modification of the peptides. 22 HA compounds were identified that were successful in modification of side chains in a range of conditions (Agniswamy *et al.*, 2008 ▸) and this included all of the compounds in the Magic Seven set. They confirmed the observation of Blundell & Johnson (1976 ▸) that methionine and histidine residues react with platinum-containing compounds, whereas cysteine residues react with mercury- and gold-containing compounds. They produced a useful web application which allows the prediction of whether a particular compound will modify the protein in a given buffer and pH, thus allowing the user to exclude compounds that are unlikely to react in a given crystallization condition (http://sis.niaid.nih.gov/cgi-bin/heavyatom_reactivity.cgi).

The Sun group also suggested that rapid screening using high concentrations (up to 10 m*M*, depending on the solubility of the compound) of a heavy metal and short soaking times (10 min) is less damaging to protein crystals than longer, slower, low-concentration soaks (Lu & Sun, 2014 ▸). However, this is likely to be dependent on the protein and the crystal, so although this could be the first method to be tried, it should not be the only one used if it does not give immediate results.

#### 2.3.5. How to select the HAs to use in practice   

In order to select a suitable HA, first the protein sequence should be checked for cysteines that might be susceptible to modification by mercury or gold, methionines for selenomethionine modification or methionines and histidines for platinum modification. Even if nothing else is attempted with HAs, it is worth at least trying derivatization with one or two mercury-containing compounds. For membrane proteins the authors’ favourite is ethylmercury thiosalicylate (EMTS), and for soluble proteins we recommend the platinum compound K_2_PtCl_4_ for a preliminary screen. For a more extensive screen, either the Magic Seven (Boggon & Shapiro, 2000 ▸) or the more comprehensive list of 22 compounds from Agniswamy *et al.* (2008 ▸) (Table 2 ▸) could be tried. For membrane proteins, the Membrane’s Eleven list (Morth *et al.*, 2006 ▸) or the Parker & Newstead (2013 ▸) list (Table 3 ▸) would be good places to start.

### 2.4. Labelled binders   

In addition to modification of the protein, it may be possible to identify a small molecule that binds to the protein that could be labelled with a heavy atom. For example, inhibitors, activators, cofactors or substrate mimics could be used for enzymes. Any small molecule that binds to the protein could potentially be either bought or synthesized with a HA attached. HA-labelled small molecules can be added to the protein prior to crystallization (co-crystallization) or soaked into existing crystals. If the ligand causes the protein to precipitate, it may be feasible to add the labelled ligand to a dilute protein sample before concentrating it in the presence of the ligand. Nucleotides bind to many proteins, and a range of brominated, iodinated, mercury- or selenomethine-derivatized nucleotides can be prepared or purchased for the phasing of such proteins. This technique has been particularly useful for work with DNA-binding proteins, since DNA molecules can be synthesized with bromine- or iodine-modified bases and then co-crystallized.

In addition to the use of labelled compounds for phasing, brominated versions of inhibitors and activators are also important for determining the binding sites of small molecules at low resolution or with low occupancy (Bagnéris *et al.*, 2015 ▸; Dong *et al.*, 2015 ▸). These derivatives may be less useful for phasing, but they will help in binding-site identification and in determining the orientation of molecules in the binding site.

### 2.5. Use of bromine and iodine as heavy atoms   

Both bromine and iodine have been used as HA derivatives to phase structures. Iodine has an anomalous signal (*f*′′) of 6.85 e^−^ at the wavelength of most home sources (Cu *K*α; 1.54168 Å), providing sufficient anomalous signal for SAD phasing. Bromine has an anomalous edge at around 0.92 Å and therefore it can be used in MAD phasing on many standard synchrotron beamlines. There are two ways to quickly insert Br or I atoms into a crystal. Crystals can be transferred into a solution containing 0.5–1.0 *M* Br^−^ or I^−^ (and all of the crystallization solution components). After a short soak (60–300 s), the crystals are cryoprotected, if necessary, in a cryoprotectant solution containing the same concentration of halide and then cryocooled prior to data collection (Dauter *et al.*, 2000 ▸). One or more halide ions can attach themselves to the protein *via* ionic interactions with the protein surface and thus form a derivative. This approach has been used extensively by the Seattle Structural Genomics Centre for Infectious Diseases to phase novel structures; 16 of the 17 structures on which this method was tested were successfully solved (Abendroth *et al.*, 2011 ▸).

An alternative approach is to modify the protein using triiodide I_3_^−^ and I_*n*_^−^ instead of I^−^, which can be less damaging to crystals (Evans & Bricogne, 2002 ▸), particularly as it is used at much lower concentrations. 1 g of KI is dissolved in 4 ml of water and 0.54 g of I_2_ is added. This stock solution gives concentrations of 0.47 *M* for I_2_ and 0.67 *M* for KI, which can then be diluted 50-fold or 100-fold into the crystallization solution to give a final concentration of around 5–10 m*M* (Evans & Bricogne, 2002 ▸). Electron-density maps show tri­iodide and higher order iodide structures in maps, and these I atoms have provided phases for successful structure determination.

In order to overcome the problem of nonspecific binding of HAs to proteins, the Sheldrick group created compounds with a series of functional groups around a ring (Beck *et al.*, 2008 ▸, 2010 ▸). They used two carboxylates and an amine group, interspersed with either I or Br atoms, around a six-carbon ring: 5-amino-2,4,6-triiodoisophthalic acid (I3C; Beck *et al.*, 2008 ▸) and 5-amino-2,4,6-tribromoisophthalic acid (B3C; Beck *et al.*, 2010 ▸). Crystals are soaked in a high concentration (0.5 *M*) of one of these compounds for 10 s and then backsoaked in a cryosolution which does not contain the HA for 5 s before being cryocooled. Backsoaking removes HAs that do not contribute to the isomorphous signal but do increase the absorption coefficient and thus the dose of X-rays absorbed by the crystal (Murray *et al.*, 2004 ▸).

### 2.6. Clusters of HAs   

There are several large polymetallic clusters of heavy metals that have been used for derivatizing crystals, notably tantalum bromide (Ta_6_Br_12_; Knäblein *et al.*, 1997 ▸) and compounds with 12 W atoms bridged by oxygen molecules (reviewed in Yonath *et al.*, 1998 ▸). These clusters are typically used to phase large molecules or complexes such as the original ribosome structures (Yonath *et al.*, 1998 ▸). They can initially be identified as a single giant feature in an electron-density map and the fine structure can subsequently be resolved. They provide strong anomalous signal and phasing power at low resolution (they have a very large *f*′′ signal), although there is frequently some rotational disorder in the way the clusters bind, so that the signal often drops off quickly at around 6 Å. For this reason, and because these large clusters can easily damage the crystal lattice, they seem to be most useful for crystals of very large assemblies which have plenty of space.

### 2.7. Derivatization with noble gases   

Noble gases such as xenon and krypton are not chemically reactive or polarizable, but they do interact with proteins by binding to hydrophobic patches or pockets on the surfaces of proteins. Pressure chambers have been designed that allow protein crystals to be exposed to noble gases at pressures of up to 2.5 MPa. It is essential to release the pressure slowly and then rapidly cryocool the crystals before the gas can diffuse away from the binding sites (Cohen *et al.*, 2001 ▸). Cells for high-pressure derivatization are available commercially, for example from Oxford Cryosystems, UK and Hampton Research, USA. If the appropriate equipment is not available in the home laboratory, it may be accessed at a synchrotron (for example at Diamond Light Source; http://www.diamond.ac.uk/Beamlines/Mx/Equipment-on-Demand/Xe-Chamber.html).

### 2.8. Useful websites describing heavy-atom derivatization of protein crystals   

There are several useful websites describing the use of heavy atoms to derivatize protein crystals compiled by Phil Jeffrey, Princeton (http://xray0.princeton.edu/~phil/Facility/heavyatompick.html and http://xray0.princeton.edu/~phil/Facility/heavy_atoms.html), Ethan Merritt at the University of Washington (http://skuld.bmsc.washington.edu/scatter/AS_periodic.html) and Bart Hazes (http://homepage.usask.ca/~pag266/bart-hazes.html).

### 2.9. Databases listing derivatization methods used to solve protein structures   

There are two databases, HAD (http://www.sbg.bio.ic.ac.uk/had/heavyatom.html; Islam *et al.*, 1998 ▸) and HATODAS II (http://hatodas.harima.riken.go.jp/; Sugahara *et al.*, 2009 ▸), giving information on HA phasing. They detail which HAs have been found to bind to which residues and the conditions for derivatization.

## 3. Safety issues   

HAs are useful because of their ability to bind to proteins, and indeed many HA compounds can attach to a range of biological molecules, including DNA. However, this property also makes most of them highly toxic to humans and many other organisms. They can be carcinogenic, teratogenic (damaging to the unborn child) and highly toxic on inhalation or on contact with skin.

It is also worth bearing in mind that uranium is radioactive as well as being toxic, so uranium derivatives should be treated with particular care, and special safety measures are usually required if a uranium-containing crystal is to be taken to a synchrotron source.

Nevertheless, if sensible precautions are put in place and followed, then it is possible to work with these compounds without risk to health or the environment. Listed below are some of the precautions that should be considered, but this is not comprehensive and is intended as a basic outline, not as a definitive safety guide.

### 3.1. Before starting work   

The local safety officer should always be consulted before starting work. It is essential to ensure that arrangements are in place to obtain, store, work with and dispose of all of the HAs that will be used. It may take some time to put these measures in place, especially if the laboratory is new and there are no protocols already established, so it would be wise to prepare for HA work well before the experiments are planned to commence.

### 3.2. Read the material data safety sheet for each compound   

All the material safety data sheets (MSDS) associated with the compounds that will be used should be read carefully. The MSDS will explain the hazards associated with a particular compound. The safety information comes in the form of R and S numbers, *i.e.* risk information and safety information. The following safety measures should always be taken, together with any additional ones advised by the safety officer in charge.

(i) The user should never come into contact with HA compounds.
(ii) A laboratory coat, two pairs of gloves and safety glasses should be worn at all times when working with HAs and gloves should be replaced regularly.
(iii) The MSDS and the manufacturer’s guide should be checked to determine which type of gloves are appropriate for use in the particular experiment. Some chemicals, *e.g.* di­methylmercury [Hg(CH_3_)_2_], can pass through latex gloves. Professor Karen Wetterhahm, a chemist who was working on the synthesis of this compound, died in 1997 from mercury poisoning, even though her only contact with the compound was when a few drops fell on her latex gloves (http://www.chm.bris.ac.uk/motm/dimethylmercury/dmmh.htm).
(iv) There are a large number of potential HA compounds available, so first use compounds with relatively low toxicity before resorting to the more dangerous ones.

### 3.3. Storage   

Any toxic or otherwise hazardous heavy-metal compound should be stored in a locked cabinet with the key located elsewhere in the care of a responsible individual (not taped to the back of the cabinet).

Only scientists who have been trained to work with heavy-metal compounds should use them and a training-record system should be in place. Small aliquots of compounds can be stored separately from the main container, so that most users only need to handle a minimum volume in one tube, *e.g.* 1 mg of compound per tube or (if the compound is sufficiently soluble and stable) a 100 m*M* stock of the compound in water stored in a locked box in a −80°C freezer. Most heavy-atom compounds can be purchased in small aliquots from vendors, so that the user only has to add water or buffer. This is perhaps an expensive approach, but it does minimize handling of these highly toxic compounds.

### 3.4. Preparing heavy-atom stocks   

In order to minimize contact with HA compounds, if working with large stocks of HA compounds it is advisable to manipulate them in a fume hood. The area should be covered with a disposable bench coat or a plastic tray to catch spills. Designated spatulas should be used only for HA compounds and should be washed into waste-disposal containers such as 50 ml falcon tubes, and all of the wash solution included with other HA waste. If there is no balance in the fume hood, then a small sample tube (*e.g.* a 200 µl tube) can be weighed, taken to the fume hood, a small amount of the compound (a few grains, 1–2 mg) added in the fume hood, the tube closed and then reweighed on the balance outside the hood. This procedure ensures that the stock bottle for the HA compound is never open outside the fume hood. The tube is then returned to the fume hood and water or buffer can be added to obtain the required concentration. If the HA is sufficiently soluble then a stock solution of 100 m*M* can be prepared, but in some cases compounds are less soluble and experimentation may be necessary, adding more liquid to find a concentration at which the compound enters into solution. Some HAs are light-sensitive, so in general it is advisable to store HA stocks in the dark. 5 µl aliquots of 100 m*M* EMTS and K_2_PtCl_4_ can be stored at −80°C with no apparent decrease in reactivity being observed over many months. This arrangement has two advantages: (i) from a safety point of view it ensures that the user has to handle a minimal amount of a toxic chemical and (ii) it greatly lowers the hurdle to actually perform the experiment because the experimenter can soak crystals with minimal preparation required.

After finishing the preparation of the HA stock solutions, all surfaces that have been in contact with HA compounds should be carefully cleaned.

### 3.5. Disposal   

Disposal of any materials that have been in contact with a HA should be through a separate HA waste stream. Solid and liquid waste, including gloves, tissues, liquids, crystallization plates and bench coat, should all go into disposal jars provided by the safety officer. Any HA-contaminated sharps should be placed into a separate HA sharps container. It is important to record how much of which element is in each jar and to dispose of the waste through an appropriate hazardous waste disposal system. It is expensive to dispose of HA waste, but it is even more expensive to dispose of unlabelled waste of unknown composition, so it is essential to record all of the metals and amounts in each container. The disposal arrangements will be different in individual laboratories, so it is essential to consult the local safety team before starting work

## 4. The practicalities of deriviatizing proteins in crystals   

### 4.1. Obtaining suitable crystals for derivatization   

A wide range of crystallization methods give crystals that are suitable for derivatization with HAs, including vapour diffusion (both sitting-drop and hanging-drop methods), under oil (McPherson & Gavira, 2014 ▸ and references therein) or even in lipidic cubic phase for membrane proteins (Landau & Rosenbusch, 1996 ▸; Caffrey *et al.*, 2012 ▸; Cherezov, 2011 ▸; Caffrey & Cherezov, 2009 ▸). In most cases it is essential to have a large number of reproducible crystals, so that a range of conditions and HAs can be tested. It is useful, although not always possible, to have a series of different crystal forms and crystallization conditions in case of problems with crystal stability and derivatization. Ideally, the crystallization condition should not contain phosphate or sulfate at high concentrations, as these tend to precipitate many HAs. Other compounds that should be avoided if possible are citrate and acetate, which can coordinate divalent metal ions, the buffers HEPES and Tris, which can react with metals, and reducing agents such as DTT and β-mercaptoethanol, which can also bind to HAs. In some cases it may be possible to grow crystals with an alternative buffer system or salt that does not bind the HA, or it may be feasible to lower the concentration of the interfering compound. Some crystals also tolerate exchange of the buffer or salt used for crystallization after the crystals have grown. If, however, these components are essential for crystallization it is still worthwhile attempting HA derivatization, as small quantities of the HA may still be available for ordered attachment to the protein even in the presence of the interfering compound.

If the crystallization solution includes the buffer cacodylate, which contains the HA arsenic, then the As atoms will absorb X-rays, causing radiation damage to the crystal. In this case it may be helpful to find an alternative stabilization buffer that that does not contain cacodylate or other heavy atoms, as this may lower the absorbed dose and thus improve the crystal lifetime in the X-ray beam.

### 4.2. Finding a suitable mother liquor/soaking solution to stabilize the crystals   

It is useful to identify a suitable mother liquor (soaking solution) in which the crystals are stable. This will contain all of the components of the crystallization condition, including salts, precipitant, buffer and additives, plus components of the protein-containing solution, including buffers, salts and ligands. It is important to pay attention to the concentrations of these components. In a vapour-diffusion experiment the components will concentrate to the point at which the precipitant concentration in the drop matches the concentration of the precipitant in the well, so the concentrations of all components should be adjusted to take this effect into account. In contrast, in an under-oil or batch experiment the concentrations of the components are diluted when the experiment is set up and will not have changed.

Membrane-protein samples usually include a detergent at a concentration above the critical micelle concentration (CMC). Detergents have a hydrophilic headgroup and a hydrophobic tail. At concentrations above the CMC they form spheres called micelles, with the tails pointing into the centre and the headgroups in contact with the surrounding aqueous solution. Membrane proteins have a partially hydrophobic surface, as the part of the protein that is normally in contact with the inside of the membrane has to be lipophilic. They are stabilized in the absence of the membrane by having the hydrophobic tails of the detergent in contact with the hydrophobic surface, effectively forming a micelle around the membrane-associated part of the protein. At concentrations below the CMC the micelles fall apart and the detergent molecules will dissociate from the protein, causing the protein to aggregate and precipitate. Therefore, it is essential that the detergent is included in the mother liquor at a concentration above the CMC.

Ideally, the concentration of detergent added to the mother liquor should match the concentration present in the protein sample. However, estimating the concentration of a detergent in a protein sample is not trivial since the detergent can concentrate when the protein is concentrated, even if a high-molecular-weight cutoff (100 or 150 kDa) filtration device is used. Hence even though there may only be twice the CMC in the final step of purification (*e.g.* 0.024% DDM in size-exclusion chromatography buffer), after concentration for crystallization the detergent concentration could be as high as 0.5–3%. It is useful to check the detergent concentration [see Strop & Brunger (2005 ▸) for a list of methods for this; the authors find the technique described in Urbani & Warne (2005 ▸) to be useful] and test different amounts of detergent if there are crystal-stability issues.

If the crystals crack, alternative mother liquors with increasing concentrations of the precipitant from 1 to 10% can be tested. Crystals can be transferred directly into a set concentration, or the precipitant concentration can be increased gradually in steps of 1 or 2% to ensure crystal stability and smaller incremental changes in the osmotic pressure as the solution constituents are exchanged. For a gradual increase in the concentration of the precipitant or other reagent, 0.5 or 1 µl solution can be added to one side of the drop and the same volume can then be aspirated from the other side of the drop followed by adding the solution with the next concentration increment to the drop, thus mixing the solutions and drawing solutions of increasing concentration over the crystal.

Instead of preparing a fresh, synthetic mother liquor, the solution from the crystallization well can be used as the mother liquor or the solution from an adjacent well. If the crystals are not stable in this, then it may be necessary to supplement it with the components of the protein sample, including detergent for membrane proteins and any ligands that have been added to the protein. Addition of protein to the soak solution may also help to stabilize the crystals.

### 4.3. Crystal handling   

Crystals can be transferred into mother liquor or HA solution in a variety of environments, using a loop to move them either to a drop of liquid on a cover slip or to a microbridge in a vapour-diffusion plate with mother liquor in the well. Alternatively, the crystals can be left in place in a crystallization plate (24-well, 96-well or 3 × 96-well plate) and the solution can be added to the drop directly. However, if the crystal supply is limited to a few crystals in a small number of drops this can be a dangerous strategy, as it may result in the loss of all of these crystals if the change in condition is not benign.

It is informative to first transfer crystals into mother liquor without HAs, soak them for some time and observe how they behave. They may crack, fall apart or dissolve, or remain intact over several hours. It is then advisable to test whether they still diffract, since even if the crystals look the same after a few hours their order may have been compromised or destroyed.

Occasionally crystals crack and then anneal, so if they look unhealthy after a few hours then they should be inspected again after 24 h just in case they have recovered.

If the crystals are stuck to the surface of a plastic plate it is better to dislodge them from the surface before adding the heavy-atom solution, as they may be more robust before encountering HAs than afterwards. A useful technique for this manipulation is to insert an acupuncture needle into the plastic of the crystallization plate next to the crystal, without touching the crystal itself. The plastic surface then deforms and often pushes the crystal off the surface.

For membrane-protein crystals grown at room temperature, the crystallization plate can be transferred to the cold room for several hours or days before attempting the derivatization and cryocooling of crystals. The technique of cooling the plates to 4°C before cryocooling is routinely used by some research groups and has allowed, for example, solution of the structure of sarcoplasmic reticulum Ca^2+^-adenosine triphos­phatase (SERCA; Sørensen *et al.*, 2006 ▸) by Poul Nissen’s group. It has also helped the authors to solve the structures of both ABCB10 (Shintre *et al.*, 2013 ▸) and ZMPSTE24 (Quigley *et al.*, 2013 ▸).

There are a few crystals that become unstable as soon as their environment is disturbed in any way, including the opening of the drop to extract crystals. In these cases it may be necessary to find alternative crystallization conditions in order to obtain a derivative. However, conditions can usually be identified under which the crystals are stable in an artificial mother liquor, at least for long enough to prepare a derivative.

### 4.4. Preparation of a heavy-atom soak solution and addition to the crystals   

Once stable crystal conditions have been established, then the HA sample in mother liquor can be prepared. The HA stock solution can be diluted to a concentration in the range 0.1–10 m*M*. A drop of the mother-liquor solution can be transferred onto a cover slip or a microbridge over a well containing crystallization solution before the crystal is fished out of its original crystallization drop with a fibre loop or micromesh and added to the drop with the HA. It is of course very important to minimize the transfer time and seal the system to prevent the crystals drying out. As an alternative to transferring the crystal, 0.5 or 1 µl of the HA-containing solution can be added directly to the crystallization drop.

If working with crystals from a 96-well plate with 20 or 60 µl crystallization solution in the well, then the HA, detergent and protein buffer can be added to the well solution, followed by addition of 0.5 µl of this solution to the drop containing the crystals. If the crystals are not stable in the well solution with added HA (and detergent), an adjacent well with higher precipitant concentration can be used (if there is one available) and the HA and buffer components added to this well before being transferred onto the crystal. If using 96-well plates with three protein positions per well, a well without crystals can be utilized by adding the HA solution to a position where there is protein but no crystal, and the crystal can then be transferred to the new position. As an alternative to adding additional solution to the crystallization drop, derivatives can also be obtained by adding a few flakes of solid HA to the drop containing the crystals. This can be achieved by dipping a thin fibre into the well solution, touching the surface of a sample of solid HA and then touching the surface of the drop containing the crystals.

### 4.5. A typical starting point for a HA soaking experiment   

As a starting point for an initial HA derivatization screen, the following could be tried.

(i) Heavy-atom concentrations of 0.1, 0.5, 1, 2 and 10 m*M*.
(ii) Times of 10, 60, 180 min and overnight.
(iii) Heavy atom: the possible choice of HA compounds is discussed in §[Sec sec2]2 above.

According to the results of these initial tests, a second round of soaking and screening crystals can be performed to refine the conditions.

### 4.6. Crystal behaviour   

Once the heavy atom has been added to the crystal, its behaviour should be observed under a microscope at intervals. Over time the crystals may crack, so their progress after 1 min, 5 min, 1 h, 3 h and even overnight should be monitored, depending on how stable they are. If the appearance of the crystals changes when the HA is added but they were stable in mother liquor, then the HA may have reacted with the protein and formed a derivative. If they have cracked or dissolved, then shorter soaks and lower concentrations of the HA can be tried to improve crystal stability. If they do not crack then it is worth cryocooling them and testing for diffraction. If there is no change in the diffraction pattern (the native and derivative data sets can be merged or an anomalous signal can be extracted), then a higher concentration for a longer time period can be tested.

If the crystals continue to crack and it has been ensured that the mother liquor and HA soak solutions are as close as possible to the conditions in the plate, higher concentrations of precipitant have been tested and the alternative handling methods described above have been tried, then first dehydrating the crystals to stabilize them and/or improve diffraction before adding the HA can be attempted. This can be achieved by suspending the crystals over a well containing increasing concentrations of precipitant, glycerol or a salt.

If it proves to be difficult to derivatize crystals after they are formed, then the protein can be derivatized prior to growing the crystals by combining lower protein concentrations with the HA and leaving the solution to incubate for some time. Prior to crystallization, the protein can be concentrated and the HA removed using repeated concentration and dilution on a filtration concentrator. However, if a membrane protein is being crystallized then the detergent may concentrate with the protein (see §[Sec sec4.2]4.2), so washing away the HA may lead to the excessive accumulation of detergent. Crystallization using similar conditions to those used for the native crystals may be successful. However, it may be necessary to screen around the original conditions rather than simply using the same condition as for the native protein, since the solubility and behaviour of the protein may be affected by the presence of the heavy atom. The use of microseeding with a seed stock derived from native crystals can also be helpful to enhance the crystallization success rate when trying to grow crystals of both HA-modified and selenomethionine-modified proteins.

### 4.7. Cryocooling crystals with or without a backsoak   

At the end of the HA soak, crystals can be cryocooled directly by being fished out with a fibre loop or micromesh and then plunged into liquid nitrogen if they are grown in a mother liquor that is also a cryoprotectant such as 30% PEG 600 or an adequate concentration of glycerol.

If a cryobuffer is required to prevent ice formation during cryocooling then the crystals can be transferred into a solution containing all of the components of the soak solution plus a suitable cryoprotectant (*e.g.* glycerol, PEG 400 or sucrose; Garman & Schneider, 1997 ▸). Ideally, this solution should be made up by replacing water with cryoprotectant rather than by diluting the original mother liquor with the cryoprotectant agent.

A key factor to consider when preparing heavy-atom-derivatized crystals is the question of ‘backsoaking’. This involves placing the crystal into a mother liquor or cryo­protectant solution without the heavy atom before cryocooling, in order to remove unbound HA. This has the advantage that any fluorescence signal detected is likely to come from a bound HA and not from the background solution. There will also be less absorption of X-rays by the crystal if there are fewer HAs within it, so there will be a lower absorbed dose and thus less radiation damage. Backsoaking works particularly well if the HA forms a covalent bond to the protein. However, backsoaking can also lead to the HA dissociating from the protein, particularly for low-affinity HAs, so it can lead to low occupancy of the HA site or a complete loss of HA binding.

In summary, for covalent binders a backsoak would be the method of choice, whereas for noncovalent binders it would be useful to collect data from crystals that have been derivatized with and without a backsoak to obtain the optimum derivative data set for a particular HA.

### 4.8. How to tell if a crystal is a derivative   

If a heavy atom covalently modifies a protein then the change in mass might be detectable either by running the protein on an SDS–PAGE gel (Boggon & Shapiro, 2000 ▸) or by using mass spectrometry to observe changes in the molecular weight of the protein (Agniswamy *et al.*, 2008 ▸; Joyce *et al.*, 2010 ▸). However, noncovalent complexes are harder to analyse using these methods.

Some heavy atoms are coloured, so the crystal will change colour when soaked, particularly when using transition metals, which often have a variety of differently coloured oxidation states, and ligand complexes. For example, Fe^2+^ is light green and Fe^3+^ is red-brown. A solution containing a HA may change colour when added to the crystallization solution or when bound to the protein in solution or in the crystal as the oxidation state of and the ligands surrounding the HA change. Many synchrotron beamlines now provide the possibility of performing a fluorescence scan on a crystal around the absorption edges of heavy atoms. There should be a peak at the position of the HA edge. Any HA present in the cryocooled sample will however give a signal. If the crystals were not backsoaked to remove unbound heavy atom then a fluorescence peak at the characteristic wavelength for that HA will be observed, but there may not be any specifically bound metal ions. In this case a fluorescence signal does not confirm that the protein is derivatized. Backsoaking removes this problem, but may also remove weakly bound HA ions.

The MicroPIXE (proton-induced X-ray emission) technique is another method for the identification of metals in crystals. HAs give a characteristic X-ray emission spectrum when excited with a proton beam (Garman & Grime, 2005 ▸) and this can be used to characterize both HA derivatives and to quantify the stoichiometric ratios of endogenous metals in protein crystals, as well as to find the degree of incorporation of selenomethionine in mammalian expression systems.

### 4.9. Data collection   

Ultimately, though, the only test of a derivative is whether it is effective for phasing the structure. Whereas this may once have been an experimentally unhelpful observation, modern synchrotrons and software have transformed the question: the intensity of synchrotron beams coupled with pixel-array detectors allow data to be collected in minutes, software pipelines [*AutoSol* (Terwilliger *et al.*, 2009 ▸), *autoSHARP* (Vonrhein *et al.*, 2007 ▸), *CRANK*2 (Skubák & Pannu, 2013 ▸), *HKL*-3000 (Minor *et al.*, 2006 ▸) and *SHELXC*/*D*/*E* (Sheldrick, 2010 ▸)] can assess each data set in minutes, and beamline robotics allow many samples to be evaluated in quick succession.

During data collection, the importance both of ensuring that the absorbed dose is low and of obtaining data with high multiplicity cannot be overemphasized. If the data are compromised by site-specific radiation damage then they may be useless for phasing and structure determination. There are protocols for exploiting dose-dependent specific damage to particular amino acids for phasing, but these methods are not widely used at present (Ravelli *et al.*, 2003 ▸). If the data indicate the presence of an anomalous signal but it does not lead to successful structure solution, this information is nevertheless strong evidence that further work on a potential derivative is worthwhile. This would include increasing the soak time and/or HA concentration or reducing radiation damage and/or increasing multiplicity in data collection in order to increase the size of the signal and thus further improve the phasing power.

A very effective way of improving the anomalous signal is to couple measurement multiplicity with reorientation of the crystal (Debreczeni *et al.*, 2003 ▸; Weinert *et al.*, 2015 ▸). This is very convenient at beamlines and in-house diffractometers that are equipped with kappa goniometers, but manual reorientation can be just as effective, albeit more tedious (Krojer *et al.*, 2013 ▸).

Once a data set has been collected, the anomalous signal can be calculated and each data set can then be tested individually (SAD) and also in combination with other available derivative and native data sets (MAD, SIR, SIRAS, MIR or MIRAS techniques) to look for HA sites. These can then be used to phase the structure and to calculate interpretable electron-density maps.

### 4.10. Characteristics of a good derivative   

Ideally, a derivative crystal would diffract to high resolution, but often the resolution and diffraction quality drop substantially when HAs are added. Derivative data sets to 3.5 Å resolution or better are often quite adequate to phase a structure. It is still worth collecting a range of data sets, even if some of them are only at 6 Å resolution or worse. These data sets may improve the phase information, particularly if several derivatives are being used.

Ideally, the crystal would have an anomalous signal to high resolution, but it is worth collecting a data set for a derivative that has a signal even to low or medium resolution, as this could contribute to successful phasing.

It is essential to limit radiation damage, particularly when recording anomalous differences, as these small differences in intensities may be obscured by changes caused by radiation-damage-induced non-isomorphism. Thus, it is better to aim for a smaller dose with lower radiation damage rather than aiming for high-resolution data by using a higher photon flux density/longer exposure and thus losing the anomalous signal.

For the isomorphous replacement method it is essential that the data sets are isomorphous, *i.e.* they have very similar unit-cell parameters and the molecules within the unit cell are similarly positioned. If the data sets are isomorphous, the differences between the data sets will come from the presence of the heavy atoms rather than from changes in the unit-cell dimensions or contents. Crick & Magdoff (1956 ▸) showed that a 0.5% change in all three unit-cell dimensions would cause a 15% change in the intensities at 3 Å resolution (Crick & Magdoff, 1956 ▸), which is more than enough to mask phase information from a derivative. In an ideal case, all of the data sets collected for native and derivatives would have identical unit-cell parameters and all of the molecules positioned in the same orientation with no site-specific radiation damage. In practice, the contents and dimensions of unit cells often vary substantially and it may be necessary to collect a range of native and HA data sets and then to cluster data sets with similar unit cells and calculate phases and maps with different combinations to obtain the best phases and interpretable maps (Liu *et al.*, 2012 ▸; Giordano *et al.*, 2012 ▸).

## 5. Case studies of soluble-protein and integral membrane-protein heavy-atom derivatization   

### 5.1. ERAP1: a straightforward example of phasing with thiomersal   

Human endoplasmic reticulum aminopeptidase 1 (ERAP1) is involved in immune and inflammatory responses. The structure determination of ERAP1 (Kochan *et al.*, 2011 ▸) represented a relatively straightforward case of HA phasing in which a structure that could not be determined by molecular replacement was solved using a single HA derivative. This protein was not easy to purify or crystallize, but a few crystals were nevertheless produced and an initial 2.7 Å resolution data set was obtained. ERAP1 has four different domains and a molecular weight of 105 kDa. The structurally similar tricorn interacting factor F3 (PDB entry 1z5h; Kyrieleis *et al.*, 2005 ▸), leukotriene A4 hydrolase (PDB entry 3b7t; Tholander *et al.*, 2008 ▸) and aminopeptidase N (PDB entry 2hpt; Addlagatta *et al.*, 2006 ▸) showed significant variation in the relative positions and orientations of the four domains. Molecular replacement gave a clear solution for only one domain, but the rest of the map was very difficult to interpret unambiguously. However, a single additional crystal (space group *P*622, unit-cell parameters *a* = *b* = 200.9, *c* = 114.3 Å, α = β = 90, γ = 120°; one molecule per asymmetric unit; 60.1% solvent content) soaked for 20 min in 10 m*M* EMTS gave a 3.4 Å resolution data set [data-collection wavelength = 0.9763 Å; anomalous multiplicity = 15; resolution (CC_anom_ ≥ 0.3) = 8 Å]. A single mercury site was identified and the data were phased using SIRAS. Following solvent flattening, an easily interpretable map was produced which could be autobuilt. From collection of the EMTS data set to deposition took only two weeks.

### 5.2. Phasing ABCB10 using multiple heavy-atom soaks   

The structure of the human mitochondrial ABC transporter ABCB10 was solved at SGC Oxford (Shintre *et al.*, 2013 ▸) using heavy-atom derivatives, since the preliminary data sets did not allow a molecular-replacement solution to be found. The initial crystals diffracted anisotropically to between 3.4 and 6.5 Å resolution (space group *P*6_2_22, unit-cell parameters *a* = *b* = 100.7, *c* = 294.2 Å, α = β = 90, γ = 120°; one molecule per asymmetric unit; 62.5% solvent content). The protein is a homodimer with three cysteines per chain. Mercury and a range of other HA derivatives were tested by growing crystals at 20°C and then transferring the crystallization plates to the cold room (6°C) for more than 24 h before handling the crystals. More than 300 potential derivative crystals were screened to find those that diffracted to beyond 6 Å resolution, and 16 data sets were collected (4 × Hg, 4 × Pt, 2 × Os, 2 × Ir, 3 × Au, 2 × Lu and 2 × Pb) with resolutions ranging from 3.5 to 6 Å. A crystal that had been briefly soaked in 10 m*M* lutetium chloride gave a higher resolution data set than the previous native data set, but was not a derivative. Mercury chloride and *p*-chloro­mercuribenzoic acid were poorly tolerated and gave a significant reduction in diffraction intensity. The only data set that showed sufficient substitution by the HA was obtained from a crystal that was soaked overnight in 1 m*M* ethylmercury thiosalicylate (EMTS). This data set extended to 4.0 Å resolution [data-collection wavelength = 0.9686 Å; anomalous multiplicity = 4, resolution (CC_anom_ ≥ 0.3) = 9 Å]. Two of the three cysteine residues were modified by Hg and this gave sufficient phase information to solve the structure by SIRAS. A data set from a crystal grown from selenomethionine-containing protein was also obtained, and the positions of the selenium peaks in a 6.5 Å resolution anomalous difference Patterson map were useful for confirmation of the chain trace.

### 5.3. ZMPSTE24 phasing: a mercury derivative and cross-crystal averaging   

ZMPSTE24 is a nuclear membrane zinc metalloprotease involved in lamin processing and premature ageing diseases such as Hutchinson–Gilford progeria syndrome. ZMPSTE24 had to be phased using HAs as there were no homologues with known structures at the time. Two crystal forms of ZMPSTE24 were grown (Quigley *et al.*, 2013 ▸) depending on whether the detergents octyl glucose neopentyl glycol and cholesteryl hemisuccinate (OGNG/CHS) or dodecylmaltoside (DDM) were used. A *P*1 form was obtained in OGNG/CHS that diffracted to 3.4 Å resolution (unit-cell parameters *a* = 61, *b* = 95.5, *c* = 131.1 Å, α = 76.7, β = 79.6, γ = 72.6°; four molecules per asymmetric unit; 61% solvent content), and a poorly reproducible *P*2_1_ form that diffracted more anisotropically to 3.6 Å resolution (unit-cell parameters *a* = 152.7, *b* = 83.9, *c* = 154.8 Å, β = 114°; four molecules per asymmetric unit; 69.9% solvent content) was also grown in DDM. The *P*1 form was used for experimental phasing because there were very few crystals of the *P*2_1_ form available. Attempts to phase the structure from the anomalous signal from an intrinsic zinc ion in the zinc metalloprotease domain were unsuccessful, since not enough phasing information could be extracted to calculate useful maps (no discernible anomalous signal was apparent from 720° of data collected from a single *P*1 crystal at the zinc edge; data-collection wavelength = 1.282 Å; anomalous multiplicity = 3). Suitable initial phases were obtained using SIRAS, again with a crystal soaked for 2 d in 1.2 m*M* EMTS, giving a data set at a resolution of 3.75 Å with 12 Hg sites in the asymmetric unit, since there were three per monomer [data-collection wavelength = 0.9686 Å; anomalous multiplicity = 3; resolution (CC_anom_ ≥ 0.3) = 7 Å]. As with ABCB10, transfer of the crystals from 20 to 4°C was essential to maintain a high degree of crystal/data-set isomorphism. Both of the crystal forms (*P*1 and *P*2_1_) had four copies of ZMPSTE24 in the asymmetric unit and allowed eightfold cross-crystal averaging to be performed, which produced high-quality maps that could be autotraced. The chain trace was validated from the positions of the five cysteine residues modified by EMTS in the heavy-atom soak and the position of the Zn atom in the zinc metalloprotease domain. ZMPSTE24 has a novel and completely unexpected fold, with seven transmembrane helices surrounding a huge water-filled chamber within the membrane and with the zinc metallo­protease domain capping one end of the chamber (Quigley *et al.*, 2013 ▸).

### 5.4. Identifying the binding site for norfluoxetine in the TREK-2 ion channel   

Another use of heavy atoms in crystal structures is for the identification of binding sites for small molecules in medium- to low-resolution data sets. Norfluoxetine (the breakdown product of fluoxetine/Prozac) is known to inhibit the K2P ion channel TREK-2 with a *k*_d_ of around 10 µ*M* (Dong *et al.*, 2015 ▸). Both soaks and co-crystallization of TREK-2 with norfluoxetine were tested, and in some data sets Y-shaped density was seen that could have been norfluoxetine, but the quality of the density was poor (resolutions of between 3.6 and 4 Å) and it was not possible to be sure whether the density was really norfluoxetine or a different molecule. A derivative of fluoxetine was therefore synthesized with a bromine on the trifluoromethyl-substituted phenoxy ring and TREK-2 was co-crystallized with this brominated norfluoxetine (Dong *et al.*, 2015 ▸). Data from crystals of TREK-2 with Br-fluoxetine [data-collection wavelength = 0.8856 Å; overall anomalous multiplicity = 6; resolution (CC_anom_ ≥ 0.3) = ∞ (*i.e.* no discernible anomalous signal in the data statistics)] gave a clear peak for the Br atom in an anomalous difference Patterson map. The Br atom was located in the fenestration which connects the vestibule below the pore filter to the inside of the membrane and it thus unequivocally confirmed the binding site for norfluoxetine in the fenestration, also indicating the orientation of this Y-shaped molecule in the density. It was thus shown that TREK-2 adopts two distinct conformations and that only one of these conformations has the norfluoxetine-binding fenestration. This distinguished the norfluoxetine-inhibited state with the helices in the ‘down’ conformation from the more active ‘up’ state of the channel, which has the helices in the raised conformation. These structures therefore explained how the state-dependent blocker norfluoxetine inhibits TREK-2 (Dong *et al.*, 2015 ▸).

## 6. Conclusion   

Heavy-atom derivatization remains a useful tool for phasing protein structures, particularly in cases where there are no known homologous structures, where there are large changes in conformation and/or the resolution is low or the chain trace is in doubt. It is not necessarily the first method to try when phasing a structure, but it is, and will continue to be, a very valuable way to obtain phase information for macromolecular structure determination.

## Figures and Tables

**Table 1:** Examples of mercury-containing compounds used to phase macromolecular structures

Methylmercury(II) acetate	MeHg(CH_3_COO)
Mercury(II) acetate	Hg(CH_3_COO)_2_
Mercury(II) chloride	HgCl_2_
Mercury(I) chloride	Hg_2_Cl_2_
Potassium tetraiodomercurate(II)	K_2_HgI_4_
4-Hydroxymercuribenzoic acid, sodium salt (POMB)	HOHgC_6_H_4_CO_2_Na
4-Chloromercuribenzoic acid, sodium salt (PCMB)	ClHgC_6_H_4_CO_2_Na
4-Chloromercuribenzenesulfonic acid, sodium salt (PCMBS)	HgC_6_H_4_SO_3_Na
Sodium ethylmercurithiosalicylate (EMTS)	C_9_H_9_HgNaO_2_S
Ethylmercury(II) chloride	C_2_H_5_HgCl
Mercury(I) acetate (dimercury acetate; DMA)	Hg_2_(CH_3_COO)_2_
Potassium tetracyanomercurate(II)	K_2_[Hg(CN)_4_]

**Table 2:** Heavy-atom compounds recommended for the derivatization of soluble proteins The Magic Seven list was derived by Boggon & Shapiro (2000 ▸) based on the information available in 2000 on successful heavy-atom phasing of soluble proteins. The more extensive list from Peter Sun’s laboratory (Agniswamy *et al.*, 2008 ▸; Joyce *et al.*, 2010 ▸; Lu & Sun, 2014 ▸) is based on studies of which heavy atoms were successful in modification of peptides in a range of buffer and pH conditions. A useful app to determine whether a particular heavy atom is likely to be successful in particular crystallization conditions can be found at http://exon.niaid.nih.gov/sis/cgi-bin/heavyatom_reactivity.cgi. Compounds that have proved to be particularly useful to the authors are shown in bold and these would be a good starting point if a limited HA screen is planned.

Magic Seven: Boggon & Shapiro (2000 ▸)		
Hg	Mercury(II) chloride	HgCl_2_
Hg	Potassium tetraiodomercurate(II)	K_2_HgI_4_
Hg	4-Chloromercuribenzenesulfonic acid, sodium salt (PCMBS)	C_6_H_4_ClHgNaSO_3_
Pt	**Potassium tetrachloroplatinate(II)**	**K_2_PtCl_4_**
Au	Potassium dicyanoaurate(I)	KAu(CN)_2_
U	Uranium(VI) oxyacetate	UO_2_(C_2_H_3_O_2_)_2_
U	Potassium uranyl fluoride	K_3_UO_2_F_5_
Agniswamy *et al.* (2008 ▸)		
Hg	Mersalyl acid	C_13_H_18_HgNO_6_
Hg	Mercury(II) acetate	Hg(CH_3_COO)_2_
Hg	Methylmercury(II) acetate	CH_3_Hg(CH_3_COO)
Hg	4-Chloromercuribenzenesulfonic acid, sodium salt (PCMBS)	C_6_H_4_ClHgNaSO_3_
Hg	Ethylmercury(II) phosphate	C_2_H_5_HgPO_4_
Hg	Methylmercury(II) chloride	CH_3_HgCl
Hg	Mercury(II) cyanide	Hg(CN)_2_
Hg	Mercury(II) bromide	HgBr_2_
Hg	**Thiomersal, thimerosal, EMTS, ethylmercurithiosalicylate**	**C_9_H_9_HgNaO_2_S**
Pt	Ammonium dinitroplatinate(II)	Pt(NH_3_)_2_(NO_2_)_2_
Pt	**Potassium tetrachloroplatinate(II)**	**K_2_PtCl_4_**
Pt	Ammonium tetrachloroplatinate(II)	NH_4_PtCl_4_
Pt	Potassium tetrabromoplatinate(II)	K_2_PtBr_4_
Pt	Potassium hexabromoplatinate(IV)	K_2_PtBr_6_
Au	Potassium tetrachloroaurate(III)	K_2_AuCl_4_
Au	Sodium tetrachloroaurate(III)	NaAuCl_4_
Au	Gold(III) chloride	AuCl_3_
Au	Potassium dicyanoaurate(I)	KAu(CN)_2_
Pb	Lead acetate	Pb(CH_3_COO)_2_
Pb	Lead nitrate	Pb(NO_3_)_2_

**Table 3:** Heavy-atom compounds suitable for integral membrane proteins The ‘Membrane’s Eleven’ HA compounds selected by Morth *et al.* (2006 ▸) were supplemented in a study by Parker & Newstead (2013 ▸) covering more recent results on HA derivatization of membrane proteins. Of the 17 compounds identified as successful for membrane proteins in the two papers, seven are found in both lists.

Category	Compound	Formula	Membranes Eleven	Parker and Newstead
Organomercurials	Methylmercury(II) acetate	CH_3_Hg(CH_3_COO)	Yes	Yes
	Ethylmercury(II) thiosalicylate	C_9_H_9_HgNaO_2_S	Yes	Yes
	4-Chloromercuribenzoic acid, sodium salt	C_7_H_5_HgNaO_3_	Yes	
	Ethylmercury(II) phosphate	C_2_H_5_HgPO_4_		Yes
	Methylmercury(II) chloride	CH_3_HgCl		Yes
	Mercury(II) chloride	HgCl_2_		Yes
Platinum	Potassium tetrachloroplatinate(II)	K_2_PtCl_4_	Yes	Yes
	Potassium hexachloroplatinate(IV)	K_2_PtCl_6_		Yes
	Potassium platinum(II) nitrate	K_2_Pt(NO_2_)_4_	Yes	Yes
Trimethyllead	Trimethyllead acetate	C_3_H_9_Pb(CH_3_COO)	Yes	Yes
Gold	Potassium gold(I) cyanide	KAu(CN)_2_	Yes	Yes
	Potassium tetrachloroaurate(III)	KAuCl_4_		Yes
Os/Ir	Osmium(III) chloride	OsCl_3_	Yes	
	Sodium iridium(III) chloride	Na_3_IrCl_6_	Yes	
Lanthanides	Ytterbium chloride (cocrystallization)	YbCl_3_	Yes	
HA cluster	Tantalum bromide	Ta_6_Br_12_	Yes	Yes
